# Effect of Diet and Oxidative Stress in the Pathogenesis of Lymphoproliferative Disorders

**DOI:** 10.3390/antiox12091674

**Published:** 2023-08-26

**Authors:** Gabriella Cancemi, Nicola Cicero, Alessandro Allegra, Sebastiano Gangemi

**Affiliations:** 1 Division of Hematology, Department of Human Pathology in Adulthood and Childhood “Gaetano Barresi”, University of Messina, Via Consolare Valeria, 98125 Messina, Italy; gabcan17@gmail.com (G.C.); aallegra@unime.it (A.A.); 2 Department of Biomedical, Dental, Morphological and Functional Imaging Sciences, University of Messina, Via Consolare Valeria, 98125 Messina, Italy; 3 Allergy and Clinical Immunology Unit, Department of Clinical and Experimental Medicine, University of Messina, Via Consolare Valeria, 98125 Messina, Italy; gangemis@unime.it

**Keywords:** lymphoma, oxidative stress, reactive oxygen species, diet, nutraceutical, antioxidant food

## Abstract

Lymphomas are a heterogeneous group of pathologies that result from clonal proliferation of lymphocytes. They are classified into Hodgkin lymphoma and non-Hodgkin lymphoma; the latter develops as a result of B, T, or NK cells undergoing malignant transformation. It is believed that diet can modulate cellular redox state and that oxidative stress is implicated in lymphomagenesis by acting on several biological mechanisms; in fact, oxidative stress can generate a state of chronic inflammation through the activation of various transcription factors, thereby increasing the production of proinflammatory cytokines and causing overstimulation of B lymphocytes in the production of antibodies and possible alterations in cellular DNA. The purpose of our work is to investigate the results of in vitro and in vivo studies on the possible interaction between lymphomas, oxidative stress, and diet. A variety of dietary regimens and substances introduced with the diet that may have antioxidant and antiproliferative effects were assessed. The possibility of using nutraceuticals as novel anticancer agents is discussed; although the use of natural substances in lymphoma therapy is an interesting field of study, further studies are needed to define the efficacy of different nutraceuticals before introducing them into clinical practice.

## 1. Introduction

### 1.1. Diet and Cancer

Cancer represents one of the main causes of death in the world [1]. It can be defined as a multifactorial disease, since both genetic mutations and environmental and lifestyle factors play a fundamental role in its genesis. Among the main factors that have a severe impact on cancer risk are diet, excessive body weight, obesity, sedentary lifestyle, tobacco use, alcohol intake, and exposure to chemical and physical agents; therefore, these factors can be intervened in prevention [2,3,4,5,6].

The tumor burden could be significantly reduced by positive behavioral changes, such as healthy diets, calorie restriction, and fasting [7,8]. A diet with a prevalent share of fruit and vegetables, low in red meat and animal fats, such as the Mediterranean diet, is known to help prevent chronic disease and cancer [9].

It is believed that diet might intervene in carcinogenesis by acting on various biological mechanisms, including inflammation, immunity, angiogenesis, growth factors, and cell cycle regulation [10]. Fruits and vegetables have been shown to have antitumor effects in different cells; they are an irreplaceable source of nutrients and health-protective substances, such as folic acid, selenium, vitamins C and E, pyridoxine and riboflavin [11,12,13]. On the contrary, red meat is associated with a higher cancer risk; this could be due to cooking at high temperatures, which determines the formation of potentially harmful DNA adducts [14]; even a high intake of heme iron could damage DNA [15], just as the high salt content present in processed meats causes inflammation and atrophy of the gastric mucosa, and therefore may determine an increased risk of gastric cancer [14]. Finally, it is also necessary to mention the carcinogenic potential of alcohol; it has a genotoxic effect on some types of cells, interfering with the DNA repair mechanisms [16,17,18].

Fasting cycles and reduced calorie intake (CR) are able to rewire cellular metabolism, protecting it from oxidative damage and extending cellular longevity. Various dietary restriction regimens can be useful in preventing the onset and spread of cancer, enhancing therapeutic response, and minimizing its harmful side effects [19]. The beneficial effects mediated by fasting and CR appear to be due to a reduction in oxidative stress, glucose levels, insulin resistance, insulin-like growth factor-1 (IGF-1), and growth hormone (GH) levels that occur in normal cells [20]. Fasting and CR have stronger impacts on cancer cells that are expressing proto-oncogenes [21]. Additionally, it has been demonstrated that CR and fasting promote cancer immunosurveillance and T-cell-mediated tumor cytotoxicity [22], modify the activity of natural killer (NK) cells [23], and may even cause immunogenic cell death. These processes lead to the initiation of autophagy, the migration of innate immune system dendritic cell precursors toward dying tumor cells that present tumor-associated antigens to cytotoxic T lymphocytes (CTLs), and a decrease in the migration of immunosuppressive Treg cells toward tumor cells [24,25]. In addition, calorie restriction can lessen the carcinogenic and metastatic potential of cancer stem cells, which is commonly thought to be the cause of tumor development and recurrence [26].

The European Prospective Investigation into Cancer and Nutrition (EPIC) study evaluated the relationship between the incidence and mortality of the most common cancers in Europe (colorectal, breast, lung, and prostate cancer) and dietary factors. This study highlighted that the Mediterranean diet represented a protective factor in the development of colorectal and breast cancer; a diet rich in fruit and vegetables played a protective action against colorectal, breast, and lung cancer, while for prostate cancer, only fruit had a protective effect. Higher fish consumption was correlated with a lower risk of breast and colorectal cancer; conversely, high consumption of red and processed meat and alcoholic beverages led to an increase in cancer risk. Finally, taking in calcium and yogurt has been shown to defend against colorectal and prostate cancer [27].

### 1.2. Oxidative Stress and Cancer

The term oxidative stress identifies an alteration of the balance between the levels of reactive oxygen species (ROS) and the activity of cellular antioxidant mechanisms in favor of the former, which can lead to potential damage [28,29,30]. ROS perform a dual role, because, at physiological concentrations, they carry out very important functions at cellular and systemic levels. They are involved in cellular responses against pathogens and participate in ordinary cellular signaling pathways [31], while excess ROS can lead to genomic and mitochondrial DNA damage, which in turn leads to molecular mutations and altered signaling pathways [1]. The genesis of ROS can be exogenous or endogenous; mitochondria, peroxisomes, and activated inflammatory cells, such as macrophages, neutrophils, and eosinophils, represent the main endogenous sources [30,32,33]. Regarding the production of exogenous ROS, these can be generated by ionizing radiation [34] or by xenobiotics (pharmaceutical or environmental chemicals) [1,30]. The main source of intracellular ROS is represented by mitochondria, which generate superoxide radicals during oxidative phosphorylation [35]. Furthermore, anion superoxide can be produced through the enzymatic activity of xanthine oxidase or during the oxidation of fatty acids [36]. Another source of intracellular ROS generation is represented by the peroxisome, which determines the formation of hydrogen peroxide and superoxide through peroxisomal oxidases, such as acyl-CoA oxidase, xanthine oxidase, and xanthine dehydrogenase. The last aforementioned is involved in the synthesis of reactive nitrogen species via purine catabolism [37,38,39].

To maintain ROS at physiological levels, cells are equipped with defense systems, referred to as antioxidants, such as glutathione (GSH), catalase (CAT), superoxide dismutase (SOD), and thioredoxin peroxidase (TPx) [40,41]. These systems ensure the balance between the positive and negative effects of ROS; when redox homeostasis fails, a condition of oxidative stress is created, which can cause harmful alterations of macromolecules, such as DNA, proteins, and lipids [31,42,43,44,45]. Oxidative stress provokes cellular lesions via two considerable phenomena: the oxidative alteration of proteins and lipid peroxidation of cell membranes [46]. Lipid peroxidation can determine injuries to both nuclear DNA and mitochondrial DNA (mtDNA), resulting in genomic changes [47].

A close relationship between the onset and progression of various human diseases, including cancer and oxidative stress, has been demonstrated by several studies [48,49,50].

**Figure 1:**
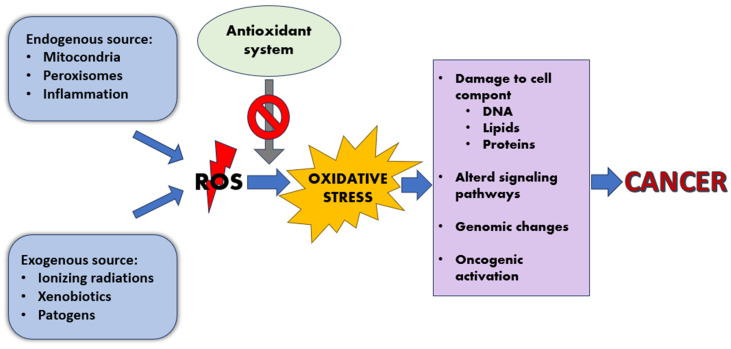
Oxidative stress’s role in the development of cancer.

In tumorigenesis, neoplastic cells exhibit metabolic changes, mainly characterized by dysregulation of glucose metabolism; due to the so-called Warburg effect, cancer cells have a tendency to resort to glycolysis for energy production to a greater extent than healthy cells, rather than aerobic cellular respiration even where oxygen is abundant. The metabolic modifications that occur during early carcinogenesis lead to the genesis of a relatively oxidative tumor microenvironment [51,52]; this is associated with mitochondrial damage and excessive superoxide production. The redox imbalance could lead to alteration of signaling pathways, genetic modifications, and oncogenic activation, laying the foundations for malignant transformation [53,54] (Figure 1).

It is well known that the permanent changes in the genetic material caused by oxidative damage represent the drive toward aging, mutagenesis, and carcinogenesis [55]. It has been demonstrated that by means of oxidative adducts, oxidative stress induces genomic damage, which is not corrected because inhibition of DNA repair mechanisms occurs, normally guaranteed by enzymes with oxidative metabolism. In this regard, ROS plays an important impact in carcinogenesis due to the genomic instability resulting from their accumulation. Indeed, high levels of ROS can act by modifying the expression of a huge number of transcription factors, activating genes involved in the regulation of second messengers, growth factors, cell cycle control factors, cytokines, and chemokines, and this self-maintains the microenvironment oxidative [1,31]. In confirmation of what has been reported, numerous oxidative damages to DNA have been observed in different types of neoplasms [56,57].

It has been uncovered by various trials that, in the long run, oxidative stress deter-mines a stable and protracted inflammatory state, which in turn is the basis of numerous chronic diseases, including cancer [31]. Mast cells and leukocytes are recruited to the damaged area during inflammation, which causes a “respiratory burst” due to increased oxygen absorption. This increases ROS release and accumulation in the damaged area [58,59]. In addition, inflammatory cells also produce cytokines, chemokines, and arachidonic acid metabolites, which recruit other inflammatory cells and increase ROS production. A vicious cycle is created, and this inflammatory/oxidative environment results in cellular damage, which, if prolonged, may lead to carcinogenesis [60]. As seen, ROS can mediate carcinogenesis either directly, through oxidation, nitration, halogenation of nuclear DNA, RNA, and lipids [61], or indirectly, through activation of various signaling pathways [62]. More than 500 different genes, including those for growth factors, inflammatory cytokines, chemokines, cell cycle regulatory molecules, and anti-inflammatory molecules, can be expressed as a result of the activation of several transcription factors by oxidative stress, including NF-κB, AP-1, p53, HIF-1α, PPAR-γ, β-catenin/Wnt, and Nrf2 [63,64]. These signaling pathways play a key role in the survival and unchecked proliferation of cancer cells and are involved in the transmission of inter or intracellular information. The mitogen-activated protein (MAP) kinase/AP-1 and NF-B pathways have been found to be the most significantly affected signaling pathways by oxidants [65]. In the MAP kinase family, which modifies gene expression by phosphorylating a variety of transcription factors, the pathway most frequently associated with the control of cell proliferation is the ERK. The activation of ERK in response to alterations in cellular redox balance has been seen [66]. Redox state affects NF-κB as well; it controls a number of genes involved in cell transformation, proliferation, and angiogenesis [67]. It has been demonstrated that NF-κB expression stimulates cell proliferation while NF-κB inhibition limits cell proliferation [68]. The mechanism by which ROS activate NF-κB is unclear, and there is a complicated interaction between NF-κB and ROS; while NF-κB can be moderately activated by mild oxidative stress, extensive oxidative stress has been seen to have the opposite effect by inhibiting it [69].

The ability of tumor cells to survive longer than normal cells is one of their distinguishing characteristics. Aberrant redox homeostasis is present in cancer cells; ROS are pro-tumorigenic; however, high levels are cytotoxic [70]. To survive the oxidative state, cancer cells increase the synthesis of nicotinamide adenine dinucleotide phosphate (NADPH) via the pentose phosphate pathway (PPP), preventing ROS levels from reaching those that would cause senescence, apoptosis, or ferroptosis [71,72]. NADPH is a critical metabolite in the reductive biosynthesis of macromolecules and is necessary for cellular antioxidant defenses; it is frequently seen that the synthesis of NADPH is enhanced in cancer cells [73]. By binding to CAT, NADPH keeps the enzyme’s antioxidant capability from being depleted by H_2_O_2_ [74]. Additionally, NADPH offers reducing equivalents for the catalytic activities of glutathione reductase (GSR) and thioredoxin reductase (TxR1 and TxR2), which are used to produce GSH from GSSG and thioredoxin-(SH)2 from thioredoxin-S2 [75].

Neoplastic cells, the tumor microenvironment (TME), and ROS interact to shape the TME, which is necessary for the advancement of cancer. Elevations in oxidative stress promote changes in the TME that sustain tumorigenesis by altering the functions of cancer-associated fibroblasts (CAFs) and tumor-associated macrophages (TAMs), and drive changes in T-cells that could suppress immune responses to cancer cells. In order to remodel the extracellular matrix (ECM), CAFs and TAMs “cooperate” with cancerous cells. This promotes tumor cell proliferation, tumor angiogenesis, immunosuppression, and tumor invasion [76]. H_2_O_2_ produced by tumor cells alters the metabolism of CAFs, increasing glucose uptake, decreasing mitochondrial activity, and producing more ROS. In turn, CAFs alter the metabolism of neighboring tumor cells, decreasing glucose uptake and increasing mitochondrial activity [77]. In addition, ROS also participate in the pro-tumorigenic, anti-inflammatory, and immunosuppressive properties of TAMs that favor tumor progression [78]. Moreover, ROS and RNS work together to inhibit T-cells and create tolerance and resistance to cytotoxic T-cells. Regulatory T (Treg) cells in the TME suppress antitumor immunity; their presence is frequently linked to a poor prognosis [79]. Cytotoxic CD8+ T lymphocytes are also present in the TME. Although CD8+ T-cells are thought to help the immune system destroy tumor cells, they frequently exhibit co-inhibitory receptors like programmed death-1 (PD-1) and are regarded as “depleted” [74].

In the field of hematology, several studies have shown the presence of a relationship between hematological malignancies and oxidative stress, for example, multiple myeloma, chronic lymphocytic leukemia, Hodgkin’s lymphoma, acute myeloid leukemia, chronic myeloid leukemia, acute lymphoblastic leukemia, myelodysplastic syndrome, and chronic myeloproliferative neoplasms [80,81,82,83,84,85,86,87,88,89,90,91,92].

### 1.3. Correlation between Diet, Oxidative Stress, and Cancer

**Figure 2:**
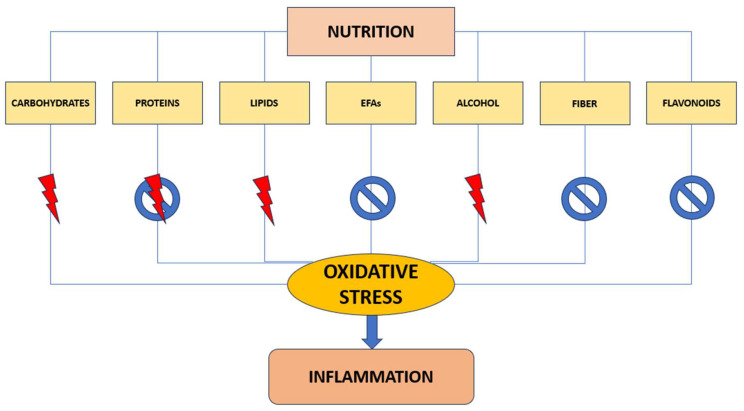
Nutrition regulates oxidative homeostasis. Lightning represents a pro-oxidant stimulus, prohibiting an antioxidant action. EFAs—essential fatty acids.

Nutrition is one of the key oxidative stress regulators in the human body; insufficient or excessive nutrient intake can disrupt oxidative homeostasis, accumulate molecular alterations in the signaling pathways of different organs, and significantly alter the cellular environment (Figure 2) [93,94]. Consequently, nutritional oxidative stress might be defined as a postprandial imbalance between pro-oxidant load and antioxidant defense as a result of insufficient or excessive nutrient intake, which may happen in cases of malnutrition or overnutrition [95,96]. Following dietary consumption of carbohydrates, proteins, and lipids, metabolic changes occur in a variety of tissues, including the adipose tissue, pancreatic beta-cells, liver, and skeletal muscle. These tissues are involved in metabolic suffering but actively interact with nutrients, leading to an increase in oxidative stress and, lastly, creating an endless cycle [93].

It has been previously demonstrated in normal subjects that after consuming glucose, mononuclear (MNC) and polymorphonuclear (PMN) leukocytes produce ROS and cause inflammation as a result of an excess of micronutrients [97]. Leukocytes can also considerably increase ROS production and inflammation after lipid intake; protein intake can also do this, although to a much smaller extent than glucose and lipid intake does [98].

Nutrition-mediated oxidative stress plays a significant role in the development of cancer. Some dietary components have a relationship with oxidative stress and, as a result, with carcinogenesis; for instance, alcohol causes biological signaling molecules to malfunction by increasing ROS production while decreasing cellular antioxidant levels [99], also causes acetaldehyde accumulation [100], and induces mitochondrial dysfunction resulting in cell death [101]. It has also been seen that a high-carbohydrate meal can result in increased oxidative stress [102]; in fact, postprandial hyperglycemia results, both in normal subjects and those with diabetes, in an imbalance in the ratio of NADH to NAD and increased non-enzymatic glycation in cells which leads to the formation of free radicals [103,104]. Regarding lipids, when caloric intake exceeds energy expenditure, excessive ROS production occurs, secondary to increased activity of the citric acid cycle [93]; in contrast, essential fatty acids (EFAs) of the omega-3 family have been shown to play a protective role from oxidative stress [105,106]. Dietary intake of fiber-rich foods would appear to protect against oxidative stress, improving mildly the indices of inflammation and oxidative stress [107,108,109]. Other nutrients that modulate cellular oxidative stress are represented by flavonides; they scavenge ROS by inactivating O_2_- radicals and stabilizing free radicals by hydrogenation or complexing with oxidant species [110,111]. Regarding protein consumption, there are conflicting opinions; according to some studies, intake of high-protein diets can cause oxidative stress, with increased risk of chronic diseases, including cancer [112,113]; other studies have not shown a correlation between protein-rich diets and long-term increase in ROS levels [114]. In addition, after a mixed meal, severe inflammatory changes were seen in normal subjects, including a decrease in inhibitor κBα (IκBα), an increase in nuclear factor κB (NF-κB), and in the inhibitory proteins p47phox subunit, IκB kinase α (IKKα), IκB kinase β (IKKβ), and plasma C-reactive protein (CRP) [115].

It can, therefore, be stated that the inflammatory and oxidative state stimulated by nutrition can alter extracellular and intracellular physiological activities. A persistent inflammatory and oxidative response happens when these dietary insults occur repeatedly, and in some cases, this can lead to various diseases. Dietary patterns that limit calories can have the exact opposite impact by ensuring oxidative equilibrium and extending the lifespan of cells [116,117].

### 1.4. Oxidative Stress in Lymphoma

Lymphomas represent a heterogeneous group of pathologies that result from a clonal proliferation of lymphocytes; they are classified into Hodgkin lymphoma (HL), 10%, and non-Hodgkin lymphoma (NHL), 90%, the latter following the malignant transformation of B, T or NK lymphocytes. NHL represents the most common hematological malignancy; in fact, it has a frequency five times greater than HL, and worldwide, it ranks 7th for prevalence in both sexes [118,119]. From a clinical point of view, lymphomas are sorted into aggressive or high-grade lymphomas and indolent or low-grade lymphomas [120]. During their evolution, progression from indolent lymphoma to aggressive lymphoma may occur [121]. Prognosis is affected by several factors, including lymphoma subtype, tumor burden, number of lymph node stations involved, and intrinsic characteristics of the subject, such as age and comorbidities [122].

Lymphomagenesis is a complex process that cannot be traced back to a single morbid event but represents the result of the interaction of various genetic and environmental factors, which, through various stages, lead to the development of a large group of different lymphoproliferative disorders [123,124]. In the first phase, there is a polyclonal proliferation related to various risk factors, including viral infections, autoimmune diseases, and immunodeficiency [125,126,127]; in the second moment, due to the involvement of tumor suppressor genes and/or proto-oncogenes, the selection of a mutant clone occurs, which has an advantage in growth and expansion compared to non-mutated cells. Inactivation of tumor suppressors and activation of proto-oncogenes are secondary to mutations, deletions, or chromosomal translocations [128,129,130]. The main translocations implicated in the genesis of lymphomas are t(14;18)(q32;q21), associated with follicular lymphoma (FL) and diffuse large B-cell lymphoma (DLBCL) [131,132]; t(8;14)(q24;q32), that is found in Burkitt lymphoma (BL) [133]; t(2;5)(p23;q35), typical of some T/NK-derived anaplastic large cell lymphomas [134]; and t(11;14)(q13;q32), described in many cases of mantle cell lymphoma (MCL) [135,136].

Confirming with the above report that the presence of genetic aberrations, in the absence of interactions with the environment, are not sufficient to result in complete malignant transformation of the cell [137], several general population studies have shown that some translocations, such as t(14,18), are also present in lymphocytes from individuals without lymphoproliferative disorders [138].

Numerous studies have tried to demonstrate the implication of oxidative stress in lymphomagenesis [139,140,141,142]. As previously mentioned, in fact, oxidative stress can generate a state of chronic inflammation through the activation of various transcription factors, thus increasing the production of proinflammatory cytokines and causing excessive stimulation of B lymphocytes in the production of antibodies and possible alterations of cellular DNA [128].

**Figure 3:**
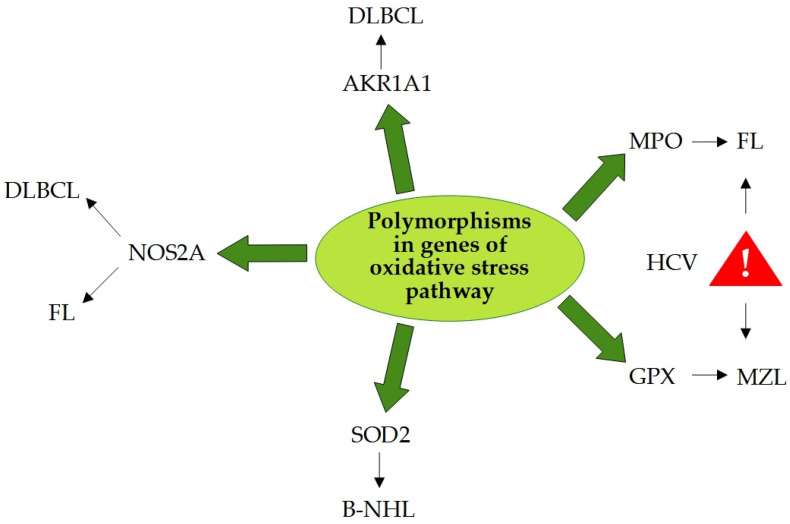
Polymorphisms in oxidative stress pathway genes increase the B-cell lymphoma risk. AKR1A1—aldo-keto reductase family 1 member A1; B-NHL—B-cell non-Hodgkin lymphoma; DLBCL—diffuse large B-cell lymphoma; FL—follicular lymphoma; GPX—glutathione peroxidase; HCV—hepatitis C virus; MPO—myeloperoxidase; MZL—marginal zone lymphoma; NOS2A—nitric oxide synthase 2A; SOD2—superoxide dismutase 2.

Genes regulating redox balance play a crucial role in lymphomagenesis; therefore, the relationship between polymorphisms in genes of the oxidative stress pathway and B-cell lymphomas was evaluated (Figure 3). Wang et al. analyzed ten oxidative stress genes (GPX, MPO, PPARG, OGG1, NOS2A, NOS3, AKR1A1, AKR1C1, SOD2, CYBA) in a multi-center study performed on patients suffering from NHL and established that polymorphisms of these genes determine an increase in ROS, which results in a greater risk of the onset of NHL [143]. It has been seen that the polymorphism (Ser608Leu) of the NOS2A gene, which codes for inducible nitric oxide synthase (iNOS), determines a greater enzymatic activity and subsequent increase in nitric oxide (NO) [144]; this polymorphism has been found in many cases of NHL, particularly in subtypes DLBCL and FL. Moreover, a statistically significant association has been identified between B-cell lymphomas and the polymorphism of manganese superoxide dismutase (SOD2 Val16Ala) [137,143]. Other studies have demonstrated an association between NHL and myeloperoxidase (MPO) and glutathione peroxidase (GPX1) polymorphisms; a correlation was seen between FL and MPO (642G > A) and between marginal zone B-cell lymphoma (MZL) and GPX1 (Ex1-226C > T) [145,146]. It has also been shown that patients carrying genetic variants of MPO and GPX1 have an additional risk of NHL if co-occurring HCV infection [147]. Furthermore, some genetic variants of AKR genes, members of the aldo-keto reductase (AKR) superfamily, have also presented an increased overall risk of NHL, most notably AKR1A1 SNP for DLBCL [145].

#### The Main Subtypes of Lymphoproliferative Disorders and Their Correlation with Oxidative Stress

A recent study conducted on patients not treated with FL evaluated the expression of redox state regulatory enzymes, such as peroxiredoxins (Prxs), enzymes that reduce H_2_O_2_ to water and molecular oxygen, and thioredoxin (Trx), an enzyme that restores the function of Prxs [148], using nitrotyrosine as a marker of oxidative damage. The results of this study suggest that elevated levels of Prxs may play a protective role in FL patients, being associated with prolonged disease-specific and overall survival [149].

There is also evidence of the involvement of oxidative stress in DLBCL; oxidative stress levels were evaluated in a study performed on 32 patients with DLBCL through Free Oxygen Radical Testing (FORT) and Free Oxygen Radical Defense (FORD), concluding that in patients with advanced DLBCL, there was an increase of free radicals and a reduced antioxidant status, suggesting that ROS play a potential role in DLBCL pathogenesis [150]. It has been seen that often, in the presence of aggressive tumors, some antioxidant enzymes promote a pro-oxidant environment; in line with this, an increased expression of GPX4 was found to correlate with a poorer prognosis in DLBCL [151]. Another example is given by the thioredoxin system (Trx), including NADPH, thioredoxin, and thioredoxin reductase, which plays a key role in redox homeostasis in all living cells, yet Trx-1 is overexpressed in many cancers and in DLBCL, conferring an advantage to lymphoma cells [152,153,154]. This makes it a promising target for the development of anticancer drugs; in fact, the negative regulation of Trx-1 lymphoma cells has been shown to sensitize to chemotherapy regimens [155].

A connection has also been demonstrated between chronic lymphocytic leukemia (CLL) and oxidative stress; in fact, it has been seen that often in CLL patients, high levels of oxidative stress biomarkers may be detected, such as malondialdehyde (MDA) and 8-oxo-20-deoxyguanosine (8-oxo-dG) [156]. Through several mechanisms, CLL cells concur in the overproduction of ROS, one of which is related to fatty acid metabolism. Specifically, it has been seen that in CLL, the Signal Transducer and Activator of Transcription 3 (STAT3) is constitutively activated, increasing the levels of lipoprotein lipase, which in turn results in enhanced and abnormal fatty acid oxidation, leading to a higher production of ROS [157]. Another mechanism is represented by the overexpression of the antioxidant enzyme hemeoxygenase-1 (HO-1), which promotes mitochondrial biogenesis, boosting mitochondrial respiration and ROS genesis, and creating a self-maintaining cycle [158]. Finally, mutations in the TP53 gene also modulate ROS levels; in fact, these are related to alterations in the genetic integrity of mitochondrial DNA, resulting in ROS overproduction and a tendency toward an oxidative state in neoplastic cells [159].

In addition, the involvement of oxidative stress has also been demonstrated in T-cell neoplasms; specifically, a loss of T-cell function and a relevant decrease in TCR signaling has been observed when these are exposed to an oxidant environment [160,161,162]. Furthermore, the increase in ROS also contributes to the inactivation of the CD45 membrane phosphatase [163]. Moreover, it has been seen that in cutaneous T-cell lymphoma, there is under-regulation of oxidative stress, resulting in increased survival of malignant T-cells, which could be used as a potential target in combination therapies, including chemotherapeutics and oxidant substances [164].

Regarding Hodgkin’s lymphoma, alteration of the redox balance in favor of a pro-oxidant state has also been demonstrated in this type of neoplasm; significant oxidative stress has been found in both RS cells and the adjacent reactive cell infiltrate. In a study conducted on lymph node samples obtained from 99 patients with Hodgkin’s lymphoma, high oxidative damage was observed; in particular, 75% of the samples showed DNA injuries, measured through the expression of 8-OHdG, and in nearly all samples, there was also an increase in nitrotyrosine, reflecting oxidative damage to proteins. Additionally, a significant expression of mitochondrial-localized antioxidant enzymes, such as manganese SOD (MnSOD) and peroxiredoxins (Prx), implicated in chemoresistance, to which a poor response to ABVD chemotherapy was correlated, was found in the most aggressive Hodgkin lymphomas [165].

Further, Morabito et al. reported increased lipid peroxidation and protein oxidation in patients with Hodgkin’s lymphoma compared with those in healthy controls. They have demonstrated a significant increment in serum levels of malondialdehyde/4-hydroxy-2,3-nonenal (MDA/HNE) and of protein carbonyl groups as parameters of lipid peroxidation and protein oxidation, respectively [166].

## 2. Lymphoma, Diet, and Oxidative Stress

Numerous studies have evaluated the relationship between lymphomas, oxidative stress, and diet (Table 1). Among them, an in vitro study using L5178Y mouse lymphoma cells exposed to β-carotene, an antioxidant found in fruits and vegetables, and catechol, a pro-oxidant and genotoxic agent. The purpose of this study was to assess whether exposure of cells to a physiologically relevant concentration of β-carotene (2 μM) resulted in a DNA damaging effect, and to evaluate the effect of the same concentration of β-carotene on DNA damage induced by different concentrations of catechol. Two different exposure protocols were used: protocol 1—L5178Y murine lymphoma cells were exposed for 3 h to β-carotene at a concentration of 2 μM and two different concentrations of catechol (0.5 or 1 mM); protocol 2—cells were pretreated for 18 h with β-carotene at a concentration of 2 μM, then were exposed for 3 h to three different catechol concentrations (0.5, 0.75 or 1 mM). β-Carotene per se was devoid of DNA-damaging effects; it appeared to reduce catechol-related oxidative DNA damage for catechol concentrations less than or equal to 0.75 mM. In contrast, it was seen that at the highest tested concentration of catechol (1 mM), β-carotene potentiated DNA damage. Thus, β-carotene had a dual effect: antioxidant for low concentrations of catechol and pro-oxidant/pro-genotoxic for high concentrations of catechol. From this, it can be inferred that β-carotene alone in ordinary conditions does not behave as a pro-oxidant/pro-genotoxic, but that it might do so in cells that are undergoing some degree of stress already [167]. Multiple investigations have demonstrated that β-carotene has antioxidant effects in vitro, and several mechanisms for this action have been hypothesized, including β-carotene-mediated scavenging or quenching of ROS and/or enhanced DNA repair [168,169,170,171,172,173]. Conversely, the mechanisms underlying this pro-oxidant activity are unknown, but they may be caused by the mutagenic effects of β-carotene cleavage products and/or the generation of oxidation products of carotenoids in an oxidative environment [174,175,176,177]. In addition, a relatively high concentration of β-carotene (20 μM) was found in other studies to increase the level of the proinflammatory cytokines IL-8 and TNF-α [178].

In another in vitro study, the phytochemical content and antioxidant activity of different parts of *Annona cherimola* fruits were characterized, followed by an investigation of the effect of these fractions on Burkitt’s lymphoma cell line (Ramos-1, CRL-1596). Aqueous, chloroform, and methanolic extracts of Annona seeds, pulp, and peel were prepared. Among all fractions, the highest concentration of polyphenols, flavonoids, and tannins was observed in methanol extracts from skin compared with other extracts from skin, pulp, and seeds, while methanol extracts from skin and pulp exhibited the greatest antioxidant activities. Lymphoma cells were treated with all extracts of Annona fractions at increasing concentrations (0, 10, 25, 50, 75, 100, 150, 200 and 300 µg/mL). The results of this study showed that water and chloroform skin extracts had no significant antiproliferative effect on Ramos-1 lymphoma cells, whereas methanol skin extract had a significant inhibitory effect on cell proliferation, with a dose-dependent effect of up to 75 μg/mL, resulting in the death of 60% of total cells. As for pulp extracts, the aqueous one produced no effect on lymphoma cells, while methanol and chloroform extracts were shown to be effective. Specifically, methanol pulp extract resulted in significant inhibition of cell proliferation with a concentration-dependent effect up to 200 μg/mL, with a response rate of about 30%; on the other hand, chloroform pulp extract had an extremely effective action, with a peak response of 90–95% at the concentration of 125 μg/mL. Aqueous seed extract also had no effect on Ramos-1 cells; methanol seed extract had limited efficacy, causing 40% inhibition at 200 μg/mL in a dose-dependent manner; in contrast, chloroform seed extract had the strongest antitumor activity, resulting in all-cell death at the greatest concentration. Although the chloroform extracts contained a relatively low concentration of polyphenols and exhibited poor levels of antioxidant activity, this fraction had the strongest antineoplastic activity, suggesting that this fruit, besides polyphenols, flavonoids, and tannins, is also rich in highly apolar substances that add to its antitumor activity [179].

Sana et al. isolated a novel phenylethanoid glycoside from *Nyctanthes arbor*-*tristis* Linn., a large ornamental shrub growing in the Indo–Pak subcontinent, called nyctanthesin A, and evaluated its anti-inflammatory, antioxidant, and anti-lymphoma activity. Compound nyctanthesin A was seen to have anti-inflammatory and antioxidant action by inhibiting in whole blood phagocytes and in isolated human polymorphonuclear cells (PMNs) MPO-related ROS production, by determining a moderate inhibition in NO production by the LPS-activated mouse macrophage cell line (J774.2), and also by reducing the production of proinflammatory cytokines, such as TNF-α in human monocytic leukemia (THP-1) cell line. Regarding the antiproliferative effects of nyctanthesin A, these were evaluated on non-Hodgkin’s B-cell lines, specifically DOHH2 cells, a follicular lymphoma cell line, and Raji cells, a Burkitt’s lymphoma cell line. In both types of lymphoma cells, inhibition of proliferation was achieved, with a predominant effect on DOHH2 cells; in contrast, the compound did not exhibit toxic effects on the proliferation of normal human fibroblast (BJ) cells. In addition, the effect of nyctanthesin A on gene and protein expression in DOHH2 and Raji cells under basal conditions and after exposure to different concentrations of nyctanthesin A (15, 30, and 60 μg/mL) was evaluated. In DOHH2 cells, exposure to nyctanthesin A resulted in significant inhibition of transcription of Bcl-2, p38 MAPK, PDL-1, and NF-κB at all concentrations used with a response greater than 90%, whereas for the COX-2 gene, there was a dose-dependent inhibition. In Raji cells, treatment with nyctanthesin A led to an inhibitory effect on the expression of COX-2, p38 MAPK, PDL-1, and NF-κB in a concentration-dependent manner, with more than 70% inhibition for COX-2 and MAPK P-38 genes; c-Myc expression was also reduced by 50% at all treated concentrations; in contrast, no inhibitory effect on Bcl-2 gene expression was observed. With regard to protein expression, an inhibitory effect of 66% for NF-κB, 51% for COX-2 and 18–30% for Bcl-2, MAPK and NF-κB was observed in DOHH2 cells exposed to 60 μg/mL nyctanthesin A, whereas in Raji cells always at the same concentration of nyctanthesin A (60 μg/mL), an inhibition of 50% for COX-2 and 18–33% for Bcl-2, p38 MAPK and NF-κB was obtained [180].

In addition to assessing the action of certain introduced substances on free radical production and subsequent increase or decrease in lymphoma risk, the correlation between alternative dietary regimens, such as intermittent fasting and cellular redox status, was evaluated. The effect of alternate-day fasting on mitochondrial ROS production, cellular oxidative status, and incidence of lymphoma in elderly mice was evaluated in a study on female OF1 mice, known to spontaneously develop aggressive lymphomas with advancing age. These mice were split into two groups at 8 months of age; one group was fed ad libitum (AL mice), while the other group was kept on alternate-day fasting (AF mice). It was seen that alternate-day fasting, when maintained for a period of 4 months, resulted in a significant reduction in the incidence of lymphoma (0% vs. 33% for controls). In addition, in AF mice, a lower oxidative state correlated with a significant increase in the SOD activity of spleen mitochondria, a reduced glutathione (GSH)/oxidized glutathione (GSSG) ratio in favor of the former, and, finally, a significant decrease in the cytosolic level of lipid peroxides was observed. The data obtained from this study suggest that alternating fasting could result in a benefit on lymphoma risk by modulating the cellular redox state [181].

Additionally, the effect of dietary restriction was evaluated in Sod1-/- mice, which are deleted for the Sod1 gene, which encodes for Cu/ZnSOD, an enzyme implicated in superoxide detoxification; these mice represent a model of accelerated aging, brought about by excess ROS, resulting in oxidative injury in various tissues. Two groups were created: a study group under dietary restriction from 2 months of age and a control group fed ad libitum. It was documented that dietary restriction had a positive effect, resulting in a 30% increase in the lifespan of Sod1-/- mice compared with controls, thus equaling that of the wild type. There was also a significant reduction in oxidative damage in Sod1-/- mice following dietary restriction, documented by the reduction of F2 isoprostanes in the liver and brain, markers of lipid peroxidation. Finally, post-mortem anatomopathological examinations showed significantly fewer pathological lesions in dietary-restricted mice than in controls, with a 5% vs. 27% incidence of lymphoma [182].

Assuming that genetic polymorphisms in genes implicated in oxidative stress may alter the role in lymphomagenesis of a diet rich in antioxidants, fruits, and vegetables, in a study of 513 female NHL patients diagnosed in Connecticut and 591 randomized controls, the correlation between these polymorphisms, fruit and vegetable intake, and the risk of NHL was evaluated. Data obtained from this study showed that, especially in DLBCL and FL subtypes, the risk of lymphoma varies with dietary intake of fruits and vegetables in the presence of particular SNPs of some oxidative stress pathway genes, such as NOS1, NOS2A, MPO, and SOD3. Specifically, an interaction of fruit and/or vegetable intake was observed with 1 SNP in the MPO gene, 1 SNP in the SOD3 gene, and 8 SNPs in NOS genes (7 in NOS1 and 1 in NOS2A). The SNP MPO (rs4401102) (CT or TT) was associated with a 1.9-fold increased risk of NHL in the group that consumed more fruits and vegetables, but not in the group that consumed less. In the low intake of vegetables group, carriers of SNP SOD3 (rs2284659) (GT or TT) had a 4.6-fold higher risk of CLL, whereas the high intake group showed a 60% reduced risk of CLL. SNP NOS1 (rs2293054) (AG or AA) resulted in a 50% reduced risk of NHL and a 60% reduced risk of FL in the high fruit and vegetable intake group, whereas the low intake group had a 2.7-fold greater risk of FL. In the low fruit and vegetable consumption group, carriers of NOS1 (rs7298903) (CT or CC) had a 1.7-fold higher risk of NHL and a 3.0-fold higher risk of FL. The risk of developing DLBCL was 60% reduced in carriers of the variant allele for NOS1 (rs545654) (CT or TT) in the low vegetable consumption group but not in the high consumption group. Considering only the intake of red vegetables, it was observed that SNPs NOS1 (rs11068446) (CT or TT), NOS1 (rs3782221) (AG or AA), NOS1 (rs7298903) (CT or CC) and NOS2A (rs3729508) (CT or TT) resulted in a 1.7–2.2-fold of DLBCL in the low red vegetable consumption group, while the high consumption group had a 30–60% reduced risk of DLBCL. In contrast, NOS1 (rs545654) (CT or TT) and NOS1 (rs12424669) (CT or TT) variants exhibited a 60% reduced risk of DLBCL in the group that consumed the fewest red vegetables and a 1.7–2.4-fold increased risk in the group that consumed the most. Finally, regarding the intake of yellow/orange vegetables, it was observed that subjects with SNPs NOS1 (rs11068446) (CT or TT), NOS1 (rs1552227) (CT or TT) and NOS1 (rs7298903) (CT or CC) showed a 2.1–2.3-fold increased risk of DLBCL in the low intake group of yellow/orange vegetables and a 40–70% reduced risk in the high intake group. This study highlights that the most significant findings involve NOS1. Thus, it can be inferred that NOS1 might play a crucial role in lymphomagenesis and that this may be subject to modification by fruit- and vegetable-based diets [45].

Given that many chemotherapeutic drugs used to treat childhood cancers increase ROS levels and that nutrition may alter the ratio of pro- to antioxidants in cells, in 32 pediatric patients with leukemia or lymphoma between the ages of 6 months and 7 years, Raber and colleagues investigated the correlation between diet, therapeutic response, and redox status in a six-month chemotherapy period. The patients were divided into two groups according to age: younger, less than 4 years of age, and older, more than 4 years of age. At baseline, there were significant differences in oxidative stress measurements between the older and younger groups; in particular, there was a more prominent oxidative state in the older group. Both groups’ superoxide levels fluctuated considerably over time, but there was not a significant distinction between groups. In addition, measures of oxidative stress were found to correlate with various foods introduced through the diet. Intracellular levels of superoxide, peroxide, and glutathione were shown to increase over the six months of chemotherapy treatment for the whole sample and were associated with the consumption of animal protein, plant protein, and total protein; glutathione was particularly observed to be positively correlated with plant protein. The findings of this investigation are consistent with earlier studies that showed distinct ROS generation after the consumption of animal and plant proteins [183].

**Table 1:** Summary of studies on lymphoma, diet, and oxidative stress.

Study Title	Object of the Study	Main Outcomes	Reference
Effect of β-carotene on catechol-induced genotoxicity in vitro: evidence of both enhanced and reduced DNA damage	In vitro Mouse lymphoma L5178Y cells	β-carotene reduces oxidative DNA damage for catechol concentrations lower than or equal to 0.75 mM, while it potentiates it for catechol concentrations greater than or equal to 1 mM.	[167]
Phytochemical profile and antioxidation activity of Anonna fruit and its effect on lymphoma cell proliferation	In vitro Burkitt lymphoma cell line Ramos-1, CRL-1596	The methanol skin extracts had the highest phenol, flavonoid, tannin content, and antioxidant activity. Methanol extracts of the skin, pulp, and seeds had a modest effect, whereas chloroform extracts of the pulp and seeds had potent effects.	[179]
Isolation and characterization of anti-inflammatory and antiproliferative compound for B-cell non-Hodgkin lymphoma from *Nyctanthes arbor-tristis* Linn.	In vitro Follicular lymphoma cell line DOHH2 and Burkitt lymphoma cell line Raji	Nyctanthesin A showed anti-inflammatory and antioxidant activity, and in lymphoma cell lines, it exhibited antiproliferative action, also acting on gene and protein expression.	[180]
Mitochondrial production of reactive oxygen species and incidence of age-associated lymphoma in OF1 mice: effect of alternate-day fasting	In vivo OF1 mice	Mice kept on alternate-day fasting had a lower oxidative state and significantly reduced incidence of lymphoma, compared with mice fed ad libitum.	[181]
Dietary restriction attenuates the accelerated aging phenotype of Sod1-/- mice	In vivo Sod1-/- mice	Dietary restriction resulted in a 30% increase in the lifespan of Sod1-/- mice compared with controls, a significant reduction in oxidative damage, and a reduction in the incidence of lymphomas.	[182]
Genetic polymorphisms in nitric oxide synthase genes modify the relationship between vegetable and fruit intake and risk of non-Hodgkin lymphoma	In vivo Female NHL patients	The risk of lymphoma varies with dietary intake of fruits and vegetables in the presence of particular SNPs, such as NOS1, NOS2A, MPO, and SOD3, with more significant results for NOS1.	[45]
Cellular oxidative stress in pediatric leukemia and lymphoma patients undergoing treatment is associated with protein consumption	In vivo pediatric leukemia and lymphoma patients	Intracellular levels of superoxide, peroxide, and glutathione increased during the six months of chemotherapy treatment and were associated with the consumption of animal protein, plant protein, and total protein; glutathione was positively correlated with the intake of plant protein.	[183]

## 3. Conclusions and Future Perspectives

Lymphomas represent common cancerous diseases; the American Cancer Society predicts that about 89,380 new cases of lymphoma will be diagnosed in the United States in 2023, of which NHL will account for 80,550 cases and HL will account for about 8830 cases [184].

Although chemotherapy is an effective treatment for lymphomas, it is often burdened by dangerous acute or chronic adverse effects, which may affect various systems, including nervous, gastrointestinal, pulmonary, skin, urogenital, cardiovascular, hematologic, and reproductive systems [185,186,187].

Nowadays, researchers are extremely interested in identifying new anticancer compounds with fewer side effects and better therapeutic outcomes [180]. A variety of naturally occurring substances in the diet, including antioxidants found in fruit and vegetables, have the potential to be employed as anticancer agents [179]. Numerous investigations in both preclinical and clinical models have shown that antioxidants could reduce the likelihood of developing cancer. As a matter of fact, epidemiological studies suggest that individuals who practice antioxidant-rich diets with high intakes of fruits and vegetables have a lower risk of developing several chronic diseases and lower mortality, compared with those who practice a diet low in fruits and vegetables [188]. Fruits and vegetables contain antioxidants and phytochemicals that can inhibit tumor progression by enhancing immune system action, decreasing oxidative state through antioxidant pathways, and regulating detoxification enzymes [189]. Antioxidant-rich nutraceuticals are attracting great interest as potential agents that could be used in cancer treatment. The scientific community is investigating numerous natural substances with antiproliferative, anti-inflammatory, and antioxidant actions, which could be candidates for making new drugs targeting hematologic malignancies and particularly lymphomas [190,191,192,193,194,195,196,197].

For this purpose, multiple compounds were examined; among these, rosemary, a medicinal plant renowned for its potential anticancer, antioxidant, and anti-inflammatory actions, as a result of the interplay between the plant’s bioactive constituents and the molecular pathways that control inflammatory processes and redox balance [198,199,200]; quercetin, a flavonoid compound found in fruits and vegetables, that executes numerous beneficial tasks by performing as an anticarcinogenic, antioxidant, anti-inflammatory, antidiabetic, and antimicrobial [201]—its capacities modify cell cycle progression, foster apoptosis, limit cell proliferation, slow the spread of metastases, and inhibit angiogenesis represent the mechanisms by which it exerts its antitumor action, observed both in vitro and in vivo [202,203]. Another polyphenol, curcumin, derived from the rhizomes of Curcuma longa, has demonstrated a wide range of therapeutic advantages towards oxidative damage, metabolic syndrome, obesity, neurological illnesses, and various malignancies. According to studies, curcumin inhibits cell development, blocks the cell cycle, and promotes apoptosis to stop the growth of various malignancies [190,204,205,206]. Even the carotenoid lycopene, found in tomatoes, pink grapefruits, pink guavas, apricots, and watermelons, is endowed with anticancer potential. Its powerful singlet oxygen quenching properties, capacity to promote the production of detoxifying/antioxidant enzymes, inhibition of cell proliferation and cell cycle progression, promotion of apoptosis, and modulation of growth factors and signal transduction pathways are the main causes of its action [207,208].

Despite several nutraceuticals having demonstrated significant anticancer potential in a variety of cancer types in vitro and in vivo, it is critical to recognize the limitations of their use in the clinical environment. One of the most important limitations is that sufficient concentrations are often not reached in the systemic circulation after ingestion of these compounds to manifest an antitumor effect [209], so further studies are needed to overcome the bioavailability barrier and achieve higher concentrations in target tissues. The use of encapsulated nanoparticles to improve the bioavailability of poorly soluble substances, like curcumin, is an example of a potential ploy [210].

In addition, it should be kept in mind that nutraceuticals are not necessarily safe for everyone. Like normal medications, they provide a physiological or pharmacological effect and may cause adverse effects in predisposed individuals and result in potential interactions with certain drugs and/or chemotherapeutics when used in combination with these [211]. Like drugs, natural substances with pharmacological activity are substrates of metabolizing enzymes, such as cytochrome P450, and may result in their suppression or induction, thereby affecting the pharmacokinetics of drugs [212].

In conclusion, although the use of mentioned above natural substances and other nutraceuticals in the therapy of lymphoma constitutes an engaging field of study, in vitro and in vivo studies are needed to define the efficacy of the different nutraceuticals, before introducing them into clinical practice.

## Data Availability

Not applicable.
